# Quantum violation of LGI under an energy constraint for different scenarios systems

**DOI:** 10.1038/s41598-023-39612-6

**Published:** 2023-08-02

**Authors:** Yuxia Zhang, Xiangguan Tan, Tianhui Qiu

**Affiliations:** 1 School of Science, Qingdao University of Technology, Qingdao, 266520 China; 2 College of Electronic and Information Engineering, Shandong University of Science and Technology, Qingdao, 266590 China

**Keywords:** Physics, Quantum physics, Statistical physics, thermodynamics and nonlinear dynamics

## Abstract

In this paper, we consider a qubit in four scenarios: with drive, without drive, and in the presence of dissipation and dephasing, to investigate the quantum violation of the Leggett–Garg inequality (LGI) in an energy constraint. In the case of the energy constraint, we find that under the coarsening measurement in reference and final resolution, the quantum violation of the LGI for the pure qubit is the most robust; on the other hand, the quantum violation of the LGI for the dephasing qubit is the most vulnerable, and the quantum violation of the LGI for driven qubit lies between that of pure qubit and dissipation qubit. Under the coarsening of measurement temporal reference, the quantum violation of the LGI for the pure qubit is more robust than that of the qubit with driven. Moreover, in the case of a qubit that is subjected to driving and is in the presence of dissipation and dephasing, the robustness of quantum violations of the LGI for these scenario systems will become vulnerable, with the driven intensity and the rate of spontaneous emission increasing, respectively, for coarsening measurement both in reference and in final resolution. In addition, in the energy constraint and the projective measurement, the LGI can attain its maximum violation value, 1.5, for the coherent dynamics; while for drive, dissipative and dephasing qubits, the LGI cannot attain the value of 1.5. For systems in the presence of dissipation and dephasing, we find that in the energy constraint, the robustness of the coarsening measurement in final resolution exhibits more vulnerable than that of the coarsening measurement in reference. And for systems with drive and without drive, the robustness of the coarsening measurement in temporal reference is the most robust, and the robustness of the coarsening of measurement final measurement resolution is the most vulnerable.

## Introduction

Since the establishment of quantum mechanics, the description of the world in quantum mechanics is very different from that in classical physics. Extrapolating the laws of quantum mechanics to the scale of everyday objects, the most famous example of it is Schrödinger’s cat, that is, the cat is in a superposition state of death and life. This example illustrates a macroscopic object being in a quantum superposition of two macroscopically different states^1^, i.e., macroscopic coherence. Such a situation runs totally counter to our intuitive understanding of how the everyday macroscopic world works. Obviously, this quantum prediction contradicts the macrorealist which asserts at any instant, a system is in any one of the available definite states. That is to say, conceptually, quantum physics is incompatible with a view of classical world. The central concepts underpinning the classical world view are codified through the notion of “macrorealism” (MR). The macrorealist view asserts that the properties of objects exist at all instants of time and are independent of the observation. In 1985, Leggett and Garg introduced the concept of macroscopic realism (macrorealism), which was described into two main assumptions^2^ (1) Macrorealism per se (MRps): A macroscopic object which has two or more macroscopically distinct states, is in a definite one of those states at any given time; (2) Non-invasive measurability (NIM): In principle, it is possible to determine which of these states the system is in without any influence on the state itself or on the subsequent system dynamics. Based on these two assumptions, Leggett and Garg formulated the Leggett–Garg inequality (LGI)^2–4^. This inequality is used to test quantum correlations in time, and is often referred to as the temporal Bell inequality^5^, which places bound on correlations between measurements for the spatially-separated systems and is based on local realism^6^. And the LGI provides a scheme for experimentally testing the compatibility between the classical world view of macrorealism and quantum mechanics. Since then, a number of experiments for violations of Leggett–Garg inequality have been performed, and the quantum behaviour can be now confirmed experimentally^7–12^. In addition, recently, there are many works to investigate influence of equilibrium and non-equilibrium environments on the LGI for many-body quantum systems^13–17^. Notably, the initial motivation of examining LGI was to test quantum superposition for macroscopic objects. Therefore, the assumption of MR is significant when one tests for the system at the macro or mesoscopic scale. However, in this paper, we consider a qubit system, for which the MR assumption has no significance as such. To be precise, we remove the macro from the MR (macrorealism), i.e., we replace MR assumption by the assumption of ’realism’ in the following. That is to say, in this paper, the assumption of ’realism’ consists of two main assumptions: realism per se (Rps) and NIM. The LGI of this paper was proposed based on the above two assumptions, which is used to test the incompatibility of the quantum-mechanical system with realism.

The projective measurement is preferable from the point of view of theory, but an accurate projective measurement is difficult to implement in certain experimental setups, which allows us to study an alternative measurement scheme: coarsening measurement. It is an inaccurate measurement. A complete measurement includes two parts: one is to set up a reference during measurement and control it; the second is to use the corresponding projection operator to make a final measurement. And the coarsening measurement can be divided into the coarsening measurement in measurement reference, the coarsening measurement in measurement time reference and the coarsening measurement in final measurement resolution^18,19^. The coarsening measurement was proposed by Kofle and Brukner^18^, which is mainly used to understand the mechanism of quantum-to-classical transition. Then, a lot of research on the coarsening measurement has been done^15,18–25^. Sumit et al.^24^ investigated the effect of the coarsening measurement time on quantum violations of macrorealism for multilevel spin systems, and found that classicality in large spin system does not emerge out of quantum mechanics under the coarsening measurement time. In Ref.^25^, they discussed non-violations of the Leggett–Garg inequality for the statistics of fluctuating work (WLG inequality), the LGI and the no-signaling-in-time (NSIT) condition in a driven two-level system under Gaussian and projective measurements. They found that the non-violation condition of the WLG inequality for the second work moment is the same as the LGI for the projective measurement, while for the Gaussian measurement, the WLG inequality cannot be violated for a wider parameter regime than the LGI.

In fact, all physical processes are subject to limitations in resources. For example, while classical capacity^26,27^ of a noiseless quantum channel^28^ may have infinite capacity for an infinite-dimensional system, this is a non-physical situation that can be overcome by optimizing capacity under energy constraints. In infinite-dimensional pure systems, the average energy constraint and the continuity property of the entanglement measure are related^29,30^, which can be quantified by considering the local von Neumann entropy. In quantum mechanics, the state of a system can be represented as a vector in a Hilbert space, which can be finite-dimensional or infinite-dimensional. Here, the energy of the system is determined by the Hamiltonian operator, which governs the time evolution of the system. The average energy of the system is then given by the expectation value of the Hamiltonian operator with respect to the state of the system. If the average energy of the system is not constrained, it is possible to find states with infinite entropy of entanglement that are very close to pure product states with vanishing entanglement. To avoid these discontinuities, additional constraints such as a bounded mean energy of the state can be imposed. By imposing such constraints, one can ensure that the entanglement measure remains continuous and well-behaved. This is important because the entanglement measure is a fundamental tool for understanding the properties of quantum systems, and its continuity is essential for making accurate predictions about the behavior of these systems. Recently, Chanda et al.^31^ have studied the average energy cost associated with the process in the LGI for the projective measurement, both in the absence of noise and in the presence of Markovian noise. Their findings indicate that, in noiseless as well as specific noisy situations, when the average energy of this process equals to the energy constraint, the LGI achieves the maximum violation^31^. However, we have not seen any report about under the energy limitation, the quantum violation of the LGI under the coarsening measurement.

In this paper, we are interested in that when an energy constraint is given, the quantum violation of the LGI under the projective and coarsening measurements. We consider a qubit in the following scenarios: pure coherent dynamics, dynamics with drive, dynamics under dissipation and dephasing. Then, in the energy constraint, we investigate the LGI for pure, driven, dissipative and dephasing qubits under the projective measurement, the coarsening measurement reference and the coarsening measurement final resolution; and we also studied an additional coarsening measurement time reference for the LGI with pure and driven qubits. We find that in the case of the energy constraint, the quantum violation of the LGI for the coherent dynamics is more robust than that of the LGI for the coherent dynamics with drive, which is more robust than that of the LGI for the dissipative qubit and the dephasing qubit, and the quantum violation of the LGI for the dephasing qubit is the most vulnerable, for coarsening measurement both in measurement reference and in final measurement resolution. And for the coarsening measurement in reference and final resolution, the robustness of quantum violations of the LGI for the qubit with drive, dissipation and dephasing, decreases with the driven intensity and the rate of spontaneous emission increasing, respectively. Furthermore, for the coarsening measurement temporal reference, we find that in the energy constraint, the robustness of quantum violation of the LGI for the driven qubit decreases as the driven intensity increases, and the robustness of this system is more vulnerable than that of the pure qubit. In Ref.^32^, they shown that for a two-level system, the maximum violation value of the LGI is 1.5. In this paper, we find that in the energy constraint, the maximum violation value (1.5) of the LGI can be attained for the pure qubit, while it cannot be attained 1.5 for the drive, dissipative and dephasing qubits, under the projective measurement. In addition, we discover that the energy change of the LGI relies on the initial state, which is different from that of the LGI. Generally, the LGI is not affected by the initial state. Moreover, for dissipative and dephasing qubits, the coarsening measurement reference exhibits more robust than that of the coarsening final measurement resolution; and for pure and driven qubits, the coarsening temporal reference is more robust than that of the coarsening measurement reference, which is more robust than that of the coarsening final measurement resolution.

## Definition and formalism

### Coarsening measurement

For a complete measurement, there are two steps involved: establishing a measurement reference and controlling it, and then using the corresponding projector to obtain a final measurement. Thus, a coarsening measurement consists of a coarsening measurement reference, a coarsening final measurement resolution^18,19^ and a coarsening measurement temporal reference^19^. We will now provide a brief introduction to the coarsening measurement. Consider a qubit observable *Q*, which can be represented by its projector as $Q=Q_{+}-Q_{-}$, where $Q_{+}=|a\rangle \langle a|$ and $Q_{-}=|b\rangle \langle b|$ with $|a\rangle$ and $|b\rangle$ being the eigenvectors of the observable *Q*. If the precision of the final measurement is coarsened, the measurement operators can be expressed in their corresponding imprecise version, as

1 $$\begin{aligned} Q_{+,\delta }= & {} (1-\delta )|a\rangle \langle a|+\delta |b\rangle \langle b|,\nonumber \\ Q_{-,\delta }= & {} (1-\delta )|b\rangle \langle b|+\delta |a\rangle \langle a|. \end{aligned}$$

The parameter $\delta$ in above expression controls the degree of the coarsening in the final measurement resolution, with the condition that $0<\delta <0.5$. Next, we show the coarsening measurement reference:

2 $$\begin{aligned} Q_{\pm , \Delta } { = \iint \limits {d\theta d\varphi {\lambda _{\Delta } }(\theta - {\theta _0})} }\lambda _{{\Delta }}(\varphi -\varphi _{0}) U {(\theta , \varphi )^\dagger } Q_{\pm ,\delta }U(\theta , \varphi ). \end{aligned}$$

Here, $U(\theta , \varphi )$ represents a unitary operator, which implies a rotation of the measurement axes about *y* axis and *z* axis:

3 $$\begin{aligned} U(\theta , \varphi )\left| 0 \right\rangle= & {} \left| {{o_n}} \right\rangle = \cos \frac{\theta }{2}\left| 0 \right\rangle + {e^{i\varphi }}\sin \frac{\theta }{2}\left| 1 \right\rangle , \nonumber \\ U(\theta , \varphi )\left| 1 \right\rangle= & {} \left| {{o_{ - n}}} \right\rangle = {e^{ - i\varphi }}\sin \frac{\theta }{2}\left| 0 \right\rangle - \cos \frac{\theta }{2}\left| 1 \right\rangle , \end{aligned}$$

where $| 0 \rangle$ and $| 1 \rangle$ are Fock states, and $\left| {{o_{\pm n}}} \right\rangle$ are the eigenvectors of $\sigma _{n}={{{\varvec{n}}}}\cdot \varvec{\sigma }$. And ${{{\varvec{n}}}}=\sin \theta \cos \varphi \,{{{\varvec{i}}}}+\sin \theta \sin \varphi \, {{{\varvec{j}}}}+\cos \theta \, {{{\varvec{k}}}}$ is a unit vector in the Bloch sphere, and $\varvec{\sigma }=\sigma _{x} \,{{{\varvec{i}}}}+\sigma _{y} \, {{{\varvec{j}}}}+\sigma _{z}\, {{{\varvec{k}}}}$ ($\sigma _{x}$, $\sigma _{y}$ and $\sigma _{z}$ are the Pauli operators). And ${\lambda _{{\Delta }} }(\theta - {\theta _0})$ and ${\lambda _{{\Delta }} }(\varphi -\varphi _{0})$ in Eq. (2) are the normalized Gaussian kernels with standard deviation ${\Delta }$ ($0<{{\Delta }}<1$), which are centered around $\theta _{0}$ and $\varphi _{0}$, respectively. Similarly, ${\Delta }$ is the degree of coarsening in the measurement reference, which determines the degree of the coarsening in measurement reference. These normalized Gaussian kernels satisfy ${\lambda _{{\Delta }} }(\theta - {\theta _0}) = \frac{1}{{\sqrt{2\pi } {{\Delta }} }}$exp$[{{ - \frac{{{{(\theta - {\theta _0})}^2}}}{{2{{{\Delta }} ^2}}}}}]$, and ${\lambda _{{\Delta }} }(\varphi -\varphi _{0}) = \frac{1}{{\sqrt{2\pi } {{\Delta }} }}$exp$[{{ - \frac{{{{(\varphi -\varphi _{0})}^2}}}{{2{{{\Delta }} ^2}}}}}]$, respectively.

Now, we introduce the coarsening measurement temporal reference^19^. Then, we consider a system whose Hamiltonian is $H(0)=\frac{1}{2}\omega \sigma _{z}$, with $\omega$ being the energy gap of the qubit. The evolution operator of system is $U(t_j, t_i)=e^{-iH \Delta t}=e^{-\frac{i}{2}\omega \sigma _{z}\tau }=e^{-\frac{i}{2}\eta \sigma _{z}}$ in the time interval between $t_{i}$ and $t_{j}$, where $\tau =\Delta t=\mid t_{j}-t_i\mid$ and $\eta =\omega \tau$. In the Schrödinger’s picture, we perform the sequential measurements of $Q(t_{i})$ and $Q(t_{j})$ at $t_i$ and $t_j$, respectively. When the temporal reference is coarsened, i.e., the unitary operation of the system is Gaussian coarsened, the corresponding joint probability for obtaining different measurement outcomes *m* and *n* at $t_i$ and $t_j$, respectively, can be written as

4 $$\begin{aligned} \begin{aligned} P_{ij, \Delta '}(m, n)&={{\textrm{Tr}}}[\sqrt{Q(t_{j})}\int \lambda _{{{\Delta }}'}(\eta '-\eta ) {{ U}(t_{j},t_{i})}\sqrt{Q(t_{i})}\rho (t_{i})\\&\quad \times {\sqrt{Q(t_{i})}}^{\dagger } {{ U}(t_{j},t_{i})}^{\dagger }{\sqrt{Q(t_{j})}}^{\dagger }d\eta '], \end{aligned} \end{aligned}$$

where $\rho (t_{i})$ is the state of system at $t_i$, and $\lambda _{{{\Delta }}'}(\eta '-\eta ) =\frac{1}{{\sqrt{2\pi } {{\Delta }'} }}$exp$[{{ - \frac{{{{(\eta '-\eta )}^2}}}{{2{{{\Delta }'} ^2}}}}}]$ is the normalized Gaussian kernel centered around $\eta$. And similarly, $0<{{\Delta }'}<1$ is the standard deviation and determines the coarsening degree of measurement reference in time as well.

### LGI

Next, we will provide a concise introduction to the LGI^2–4^. We consider an dichotomic observable *Q*, and it can only take on two values $$\pm 1$$. When this observable is measured, it will give one of these two values. Two dichotomic measurements $$ Q(t_{i})$$ and $$ Q(t_{j})$$ are performed at $$t_{i}$$ and $$t_{j}$$, respectively, where $$i, j = 0, 1, 2$$ and $$i <j$$. Then, the LGI is shown as

5 $$\begin{aligned} K_{LG}=C_{01}+C_{12}-C_{02}\le 1, \end{aligned}$$

where $C_{ij}=\langle Q_m(t_{i})Q_n(t_{j})\rangle =\sum _{m, n=\pm 1}{m}{n}P_{i j}(m,n)$ is the temporal correlation function for the dichotomic measurement operator *Q*. Here, $P_{ij}(m,n)$ is the probability of obtaining outcomes *m* and *n* at $t_i$ and $t_j$, respectively. And $m,n=\pm 1$ are measurement outcomes. If the LGI, i.e., $K_{LG}$ referenced in Eq. (5), is violated, it means that at least one of realism assumptions, i.e., Rps and NIM, is false. In addition, the maximum violation value of the LGI for a two-level system, is 1.5^32^.

### Energy change of LGI and energy constraint

**Figure 1:**
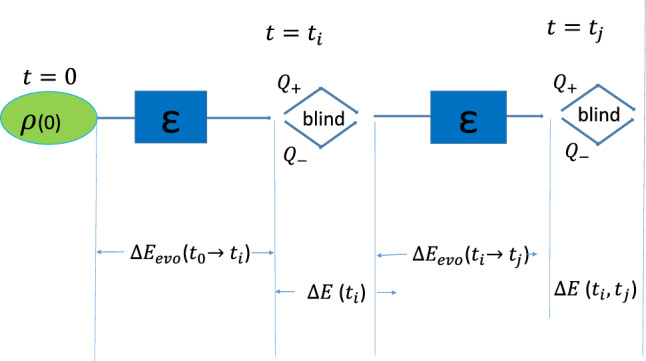
Schematic diagram for the change of energy $\Delta E_{ij}$. $Q^{\pm }$ represent general dichotomic measurements that can yield either $+1$ or $-1$ as outcomes. $\varepsilon$ denotes the system propagator $\rho \rightarrow \varepsilon (\rho )$.

Next, we discuss the energy change associated with the process contingent to temporal correlations involved in $K_{LG}$ of Eq. (5). In this experimental scenario, the energy changes for the system contain the energy changes of sequential measurements and evolution. For simplicity, we use the energy change, $\Delta E_{ij}$, related to the process $C_{ij}$ as an illustration. The change in energy $\Delta E_{ij}$ of the system in the whole process $C_{ij}$, contains (see Fig. 1 for the schematic diagram, which is an adapted version of Fig. 1 in Ref.^31^):

I. the energy change during the evolution from $t=t_{0}=0$ to $t=t_i$, $\Delta E_{evo}(t_0\rightarrow t_i)$;
II. the change of energy before and after blind measurements at $t=t_i$, $\Delta E(t_{i})$;
III. the alteration in energy throughout the process of evolution from $t=t_i$ to $t=t_j$, $\Delta E_{evo}(t_i\rightarrow t_j)$;
IV. the energy change before and after the blind measurement at $t=t_j$, $\Delta E(t_{i}, t_j)$.

Therefore, $\Delta E_{ij}=\Delta E_{evo}(t_0\rightarrow t_i)+\Delta E(t_i)+\Delta E_{evo}(t_i \rightarrow t_j)+\Delta E(t_{i}, t_j)$. Here, the change of energy $\Delta E_{evo}(t_i \rightarrow t_j)$ during the evolution from $t=t_i$ to $t=t_j$ can be written as

6 $$\begin{aligned} \Delta E_{evo}(t_i \rightarrow t_j)={{\textrm{Tr}}}[H(t_j)\rho (t_j)]-{{\textrm{Tr}}}[H(t_i)\rho (t_i)], \end{aligned}$$

where $\rho (t_i)$ is the density matrix at $t=t_i$, and $\rho (t_j)$ is the density matrix at $t=t_j$ which is evolved from $\rho (t_i)$. And $H(t_i)$ and $H(t_j)$ are Hamiltonians at $t=t_i$ and $t=t_j$, respectively. Thus, $\Delta E_{evo}(t_0\rightarrow t_i)$ and $\Delta E_{evo}(t_i\rightarrow t_j)$ in $\Delta E_{ij}$ can be derived from Eq. (6). And the change of energy $\Delta E(t_i)$ before and after blind measurements at $t_i$, can be expressed as

7 $$\begin{aligned} \Delta E(t_i)={{\textrm{Tr}}}[H(t_i) (\Sigma _{m}Q_{m}\rho (t_i)Q_{m}^{\dagger })]-{{\textrm{Tr}}}[H(t_i)\rho (t_i)]. \end{aligned}$$

From Eq. (7), we can obtain the change of energy $\Delta E(t_i)$ and $\Delta E(t_{i}, t_j)$ in $\Delta E_{ij}$. Therefore, the energy change associated with the process involved in $K_{LG}$ can be given by

8 $$\begin{aligned} \Delta E=\frac{1}{3}\sum _{t_{i},t_{j}}\Delta E_{ij} \qquad \qquad (i,j=0,1,2, and\quad i<j). \end{aligned}$$

In this paper, we suppose that the energy constraint condition is when the energy change of the LGI with the projective measurement is equal to the negative of the energy of the initial state. That is to say, we define the energy constraint as

9 $$\begin{aligned} \Delta E_{constraint}=\Delta E=-{{\textrm{Tr}}}[\rho (0)H(0)], \end{aligned}$$

where $\rho (0)$ is the density matrix at $t=0$, and *H*(0) is the Hamiltonian at $t=0$. It is noted that this definition of the energy constraint will be used throughout this paper. In the following, we will discuss the quantum violation of the LGI for the projective measurement and the coarsening measurement in the case of the energy constraint.

## A prue qubit

Firstly, we consider coherent dynamics, whose Hamiltonian is described as $H(0)=\frac{1}{2}\omega \sigma _{z}$. And the initial state of the system at $t_0 = 0$ is denoted as

10 $$\begin{aligned} \rho (0)=\frac{1-\alpha }{2}|0\rangle \langle 0|+\frac{1+\alpha }{2}|1\rangle \langle 1|, \end{aligned}$$

where $0\le \alpha \le 1$. The evolution operator of system is $U(t_j, t_i)=e^{-\frac{i}{2}\omega \sigma _{z}\tau }$ in the time interval between $t_{i}$ and $t_{j}$, where $\tau \in (0, \pi ]$. We suppose that the measurement of a dichotomic observable is equivalent to a measurement of the Bloch sphere component along a direction of $\theta$ and $\phi$, i.e., $Q(\theta ,\phi )=Q_{+}(\theta ,\phi )-Q_{-}(\theta ,\phi )$. Here,

11 $$\begin{aligned} Q_{+}(\theta ,\phi )= & {} |a(\theta ,\phi )\rangle \langle a(\theta ,\phi )|,\nonumber \\ Q_{-}(\theta ,\phi )= & {} |b(\theta ,\phi )\rangle \langle b(\theta ,\phi )|, \end{aligned}$$

where $$|a(\theta ,\phi )\rangle $$ $$=\cos \frac{\theta }{2}|0\rangle +e^{i\phi }\sin \frac{\theta }{2}|1\rangle $$ and $$|b(\theta ,\phi )\rangle =\sin \frac{\theta }{2}|0\rangle -e^{i\phi }\cos \frac{\theta }{2}|1\rangle $$ ($$\theta \in [0, \pi )$$, $$\phi \in [0, 2\pi )$$). And then, two projective measurements $$ Q(t_{i})$$ and $$ Q(t_{j})$$ are performed at $$t_{i}$$ and $$t_{j}$$, respectively, with $$t_{i}<t_{j}$$. Now, we study the change in energy of the LGI in Eq. (8) for the pure qubit in the case of the projective measurement. From Eqs. (8), (10) and (11), the change in energy of the LGI associated with the process involved in $$K_{LG}$$ under the projective measurement for the pure qubit, can be expressed as

12 $$\begin{aligned} \Delta E=\frac{\alpha \omega }{24} \left[ 6 \sin ^2\theta (\cos 2 \theta +3)-\sin ^2 2 \theta (2 \cos \omega \tau +\cos 2\omega \tau )\right] . \end{aligned}$$

From above expression, it can be found that the energy change of the LGI for the pure qubit depends on the initial state, which is different from that of the LGI. In general, the LGI is independent of the initial state. And it can be found from Eq. (12) that when $\theta =\frac{\pi }{2}$, $\Delta E=\frac{ \alpha \omega }{2}=-{{\textrm{Tr}}}[\rho ({0})H({0})]=\Delta E_{constraint}$. In other words, when $\theta =\frac{\pi }{2}$, the energy change of the LGI with the projective measurement is equal to the negative of the energy of the initial state. And in this chapter, we will use this energy constraint condition, i.e., $\theta =\frac{\pi }{2}$, to study the quantum violation of the LGI for the pure qubit system. Then, we will discuss quantum violation of the LGI for the projective measurement, in the energy constraint. For the projective measurement, the corresponding probability $P_{ij}(m, n)$ in Eq. (5) satisfies

13 $$\begin{aligned} P_{ij}(m, n) ={{\textrm{Tr}}}[Q_n(t_{j})U(t_{j},t_{i})Q_m(t_{i})U(t_{i},{ t_{0}})\rho (0) {{ U}}(t_{i},t_{0})^{\dagger } {Q_m(t_{i})^{\dagger }}{{ U}(t_{j},t_{i})^{\dagger }}Q_n(t_{j}) ^{\dagger }], \end{aligned}$$

which is obtaining outcomes *m* and *n* at $t_i$ and $t_j$, respectively, with $m, n=\pm 1$ being measurement outcomes. From Eqs. (5), (10), (11) and (13), in the case of the energy constraint (i.e., $\theta =\frac{\pi }{2}$), $K_{LG}$ of Eq. (5) can be obtained as $K_{LG}=2 \cos \omega \tau -\cos 2 \omega \tau$, and we find that the LGI can be violated in the case of $0<t<\frac{\pi }{2\omega }$. And if $\theta =\frac{\pi }{2}$ and $t=\frac{\pi }{3\omega }$, the LGI can realize its maximum value, 1.5.

Now, we discuss the effects of the coarsening measurement reference on the LGI, when the energy cost is given by $\Delta E_{constraint}$. If the measurement reference ($\delta =0$ and $\Delta \ne 0$) is coarsened, and the corresponding probability in Eq. (13) can be rewritten as

14 $$\begin{aligned} P_{ij, \Delta }(m, n) ={{\textrm{Tr}}}[\sqrt{Q_{n, \Delta }(t_{j})}{{ U}(t_{j},t_{i})} \sqrt{Q_{m, \Delta }(t_{i})}U(t_{i},t_{0})\rho ({0}) {{ U}}(t_{i},t_{0})^{\dagger } {\sqrt{Q_{m, \Delta }(t_{i})}}^{\dagger }{{ U}(t_{j},t_{i})^{\dagger }}{\sqrt{Q_{n, \Delta }(t_{j})}}^{\dagger }], \end{aligned}$$

for obtaining outcomes *m* and *n* at $t_i$ and $t_j$, respectively. From Eqs. (1)–(3), (5), (10) and (14), the LGI in the energy constraint under the coarsening measurement reference can be described as

15 $$\begin{aligned} K_{LG, \Delta }= -e^{-2 \Delta ^2} (\cos 2 \omega \tau -2 \cos \omega \tau ). \end{aligned}$$

We define the value of making $K_{LG, \Delta }=1$ as a critical value of the LGI, which is denoted as $\Delta _{critical}$. In the following, we assume $\tau =\frac{\pi }{4\omega }$ (in this case, the LGI under the projective measurement is violated), to study the critical value of the LGI. Then, the critical value of the LGI from Eq. (15) under the coarsening of measurement reference can be found, i.e., $\Delta _{critical}=0.4162$. When $0<\Delta \le \Delta _{critical}$, i.e., when $0<\Delta \le 0.4162$, the LGI for the coarsening of measurement reference can be violated.

We then consider quantum violation of the LGI for the coarsening final measurement resolution ($\delta \ne 0$ and $\Delta = 0$) in the energy constraint. The LGI in the coarsening measurement resolution and the energy constraint, can be derived from Eqs. (1), (5), (10), and (14). Similarly, the critical value of the LGI is defined as the value at which $K_{LG, \delta }=1$, which is denoted as $\delta _{critical}$. And we also suppose $\tau =\frac{\pi }{4\omega }$ to study quantum violation of the LGI. Then, we find that when $\tau =\frac{\pi }{4\omega }$, the critical value of the LGI under the coarsening of measurement resolution is $\delta _{critical}=0.0795$. That is to say, the violation of the LGI can be reached, when $0<\delta \le 0.0795$.

Next, when a measurement reference in time is coarsened, from Eqs. (4), (5) and (10), the LGI in the energy constraint can be obtained as

16 $$\begin{aligned} K_{LG, \Delta '}= 2 e^{-\frac{{\Delta '} ^2}{2}} \cos \omega \tau -e^{-2 {\Delta '} ^2} \cos 2 \omega \tau . \end{aligned}$$

Similarly, the critical value of the LGI is denoted as $\Delta '_{critical}$, which is defined as the value at which $K_{LG, \Delta '}=1$. And we also suppose $\tau =\frac{\pi }{4\omega }$ to study quantum violation of the LGI. From Eq. (16), the critical value of the LGI under the coarsening measurement time reference can be obtained as $\Delta '_{critical}=0.8325$. The LGI can be violated, if $0<\Delta '\le 0.8325$.

In short, we find that the energy change of the LGI depends on the initial state. This is different from that of the LGI, because the LGI is independent of the initial state. In addition, we find that in the situation of the energy constraint, the maximum violation value, 1.5, of the LGI can be realized under the projective measurement. For the coarsening measurement reference, the coarsening final measurement resolution and coarsening time reference, the critical values of the LGI are $\Delta _{critical}=0.4162$, $\delta _{critical}=0.0795$ and $\Delta '_{critical}=0.8325$, respectively. Comparing these different critical values, we find that the coarsening measurement temporal reference is more robust than that of the coarsening measurement reference, which is the more robust than that of the coarsening final measurement resolution. That is to say, as the coarsening degree increases, the non-violation of the LGI under the coarsening measurement of final resolution can prior to occur than that of the coarsening measurement reference, and the non-violation of the LGI under the coarsening measurement reference can prior to occur than that of the coarsening measurement temporal reference.

##  A qubit with driven

Next, we consider a driven two-level system. Its Hamiltonian is time-dependent and is given by

17 $$\begin{aligned} H(t)=\frac{1}{2}\omega \sigma _{z}+\frac{g}{2}[\sigma _{x}\cos \omega t+\sigma _{y}\sin {\omega t}], \end{aligned}$$

where $g\in [0, \omega ]$ is the driving intensity quantifying the coupling to the external field, and $\omega >0$ is the free frequency of the two-level system and also the driving frequency. The system evolves under the unitary operator $U(t,0)=\overleftarrow{T}\exp [-i\int _{0}^{t}d\tau H(\tau )]$, where $\overleftarrow{T}$ is a time-ordering operator. The unitary operator can be expressed as^33,34^

18 $$\begin{aligned} U(t,0)=\left( \begin{array}{cc} e^{-\frac{1}{2} i t \omega } \cos \left( \frac{g t}{2}\right) &{} -i e^{-\frac{1}{2} i t \omega } \sin \left( \frac{g t}{2}\right) \\ -i e^{\frac{i t \omega }{2}} \sin \left( \frac{g t}{2}\right) &{} e^{\frac{i t \omega }{2}} \cos \left( \frac{g t}{2}\right) \\ \end{array} \right) . \end{aligned}$$

This unitary operator satisfies $U^{\dagger }(t,0)U(t,0)=I$ and $U(t_{j},t_{i})U(t_{i},0)=U(t_{j},0)$ with $i <j$.

Next, from Eqs. (8), (10), (11), (13), (17) and (18), we obtain change in the energy of the LGI for the projective measurement, which is given by

19 $$\begin{aligned} \Delta E=\frac{1}{24} \alpha \left[ g \sin 2 g \tau \sin ^2(\phi - \omega \tau ) \sin (2\phi -4 \omega \tau )+2 g \sin g\tau \sin (2\phi -2\omega \tau ) \cos ^2(\phi -2 \omega \tau )+12 \omega \right] . \end{aligned}$$

It can be found from above expression that when $\theta =\frac{\pi }{2}$, $\tau =\frac{\pi }{4\omega }$ and $\phi =\frac{\pi }{4}$, $\Delta E=\Delta E_{constraint}=-{{\textrm{Tr}}}[\rho ({0})H({0})]$. In this chapter, we will use this condition of energy constraint to discuss the quantum violation of the LGI for the system with driven. Then, from Eqs. (5), (10), (11), (13), (17) and (18), the LGI with the projective measurement under the energy constraint can be obtained (see Supplementary Information). We find that in the energy constraint, the LGI for the projective measurement, can be violated when $g\le 0.8902\omega$. In addition, in the previous chapter, we mentioned that for a two-level system, the maximum violation value of the LGI is 1.5^32^. In this chapter, we find that with the energy constraint, the LGI for the driven qubit cannot realize the maximum violation value 1.5.

Next, for the way of measurement being coarsening measurement, i.e., coarsening measurement reference ($\delta =0$ and $\Delta \ne 0$), coarsening measurement final resolution ($\delta \ne 0$ and $\Delta = 0$) and coarsening measurement temporal reference, the LGI for the energy constraint can be obtained from Eqs. (1)–(5), (10), (14), (17) and (18), which is shown in Supplementary Information. Similarly, we also define the value where the LG function equals to 1, as a critical value of the LGI. Then, in the energy constraint, the critical values of the LGI under the coarsening measurement in reference, in final resolution and in time reference, for $g=0, 0.05\omega , 0.1\omega , 0.2\omega , 0.3\omega , 0.4\omega , 0.5\omega , 0.6\omega , 0.7\omega , 0.8\omega , 0.9\omega , \omega$, are summarized in Table 1. It can be seen from Table 1 that the critical value of the LGI in the energy constraint, decreases with the driving intensity g increasing, when the measurement is the coarsening measurement at reference, the coarsening measurement at final resolution and the coarsening measurement at temporal reference. In other words, the robustness of quantum violation of the LGI decreases with increasing value of g for the coarsening measurement at reference, at final resolution and at temporal reference. In addition, comparing critical values of the LGI under the coarsening measurement (see Table 1), we find that the coarsening in measurement reference is more robust than the coarsening in final measurement resolution, while it is more vulnerable than the coarsening in temporal reference. Then, in the energy constraint, comparing the LGI for the driven qubit with the pure qubit under the coarsening reference, we find that the LGI for the pure qubit can be violated only if $\Delta \le 0.4162$, which is the same as that of the LGI for the driven qubit for the driving intensity $g=0$. We have shown that as the driving intensity g increases, the critical value of the LGI for the driven qubit decreases (see Table 1). Therefore, when the LGI for the pure qubit is not violated, the LGI for the driven qubit can not be violated too for any g. Moreover, for the coarsening measurement in final resolution, the violation condition of the LGI for the pure qubit is $\delta \le 0.0795$, which is the same as that of the LGI for the driven qubit with the driving intensity $g=0$. The critical value of the LGI for the driven qubit decreases with g increasing, which is similar to the LGI for the driven qubit in the coarsening measurement reference. Thus, for the coarsening measurement in final resolution, when the LGI for the driven qubit is violated for any g, the LGI for the pure qubit must be violated too. Furthermore, concerning the coarsening measurement at temporal reference, it was observed that the critical value of LGI for the pure qubit is $\Delta '_{critical}= 0.8325$, which is identical to the critical value obtained for the driven qubit at zero driving intensity ($g=0$). And the critical value of the LGI observed for the driven qubit in the coarsening measurement time reference decreases as the driving intensity *g* increases. Consequently, in the context of the coarsening measurement at time reference, whenever the LGI is violated for the driven qubit at any value of *g*, it is guaranteed that the LGI will also be violated for the pure qubit. In summary, under the energy constraint, the LGI for the pure qubit can be violated for a wider parameter than the LGI for the driven qubit with any g, for the measurement of coarsening at the reference, at the final resolution and at time reference. Furthermore, for the driven qubit, we find that the robustness of the coarsening measurement time reference is the most robust, and the robustness of the coarsening final measurement resolution is the most vulnerable, which is similar to that of the pure qubit. In addition, we find that in the energy constraint and the projective measurement, the LGI can attain its maximum violation value 1.5 for the coherent dynamics, while for the drive qubit, the LGI cannot.

**Table 1:** The critical values of the LGI in the energy constraint (i.e., $\theta =\frac{\pi }{2}$, $\tau =\frac{\pi }{4\omega }$ and $\phi =\frac{\pi }{4}$) for the dynamics under driven (with the coarsening measurement reference, the coarsening final measurement resolution and the coarsening time reference), the dynamics under dissipation and dephasing (with the coarsening measurement reference and the coarsening final measurement resolution), for $g/\gamma =0, 0.05\omega , 0.1\omega , 0.2\omega , 0.3\omega , 0.4\omega , 0.5\omega , 0.6\omega , 0.7\omega , 0.8\omega ,$ $0.9\omega , \omega$, respectively.

$g/\gamma$	Dynamics with driven			Dynamics under dissipation		Dynamics under dephasing	
	$\Delta _{critical}$	$\delta _{critical}$	$\Delta '_{critical}$	$\Delta _{critical}$	$\delta _{critical}$	$\Delta _{critical}$	$\delta _{critical}$
0	0.4162	0.0795	0.8325	0.4162	0.0795	0.4162	0.0795
$0.05\omega$	0.4156	0.0793	0.8321	0.3919	0.0712	0.3078	0.0451
$0.1\omega$	0.4136	0.0786	0.8308	0.366	0.0627	0.1272	0.008
$0.2\omega$	0.4056	0.0758	0.824	0.3078	0.0451	No	No
$0.3\omega$	0.3919	0.0712	0.8069	0.2355	0.0269	No	No
$0.4\omega$	0.3719	0.0645	0.7647	0.1272	0.008	No	No
$0.5\omega$	0.3444	0.0559	0.6605	No	No	No	No
$0.6\omega$	0.3074	0.045	0.4809	No	No	No	No
$0.7\omega$	0.2569	0.0319	0.3278	No	No	No	No
$0.8\omega$	0.1822	0.0163	0.1988	No	No	No	No
$0.9\omega$	No	No	No	No	No	No	No
$\omega$	No	No	No	No	No	No	No

It is noted that the “No” in the table below represents that we cannot find any parameter for $\Delta \in (0, 1)$ or $\delta \in (0, 0.5)$ to make the LGI violated. In other words, no matter what values of $\Delta$ ($\Delta \in (0, 1)$) and $\delta$ ($\delta \in (0, 0.5)$) take, the LGI is not violated.

## Open system

Previously, we studied the quantum violation of the LGI in an energy constraint for the closed system. Actually, quantum systems inevitably suffer from unwanted interactions with environment. Next, we introduce the interaction of environment to investigate the characteristics of the quantum violation of the LGI in an energy constraint. Different from the closed system discussed in the previous sections, the time evolution of the open system in general cannot be described by a unitary time evolution. The dynamics of this system is typically characterized by a quantum master equation, which, in this scenario, is commonly formulated using the Lindblad form master equation, and can be expressed as

20 $$\begin{aligned} \frac{d\rho }{d t}=-i[H, \rho ]+\sum _{k}\left[ 2 L_{k} \rho L_{k}^{\dagger }-L_{k}^{\dagger } L_{k} \rho -\rho L_{k}^{\dagger } L_{k}\right] . \end{aligned}$$

Here $L_{k}$ is the Lindblad operator, which is the coupling of the system with its environment, and the Hamiltonian *H* denotes the coherent part of the dynamics. It is noted that because in general the time evolution for the open system is not denoted by a unitary time evolution, in this chapter, we do not study the LGI with the unitary operation of the system being Gaussian coarsened, i.e., coarsening measurement time reference.

### A qubit with dissipation

We consider the first case, that the Hamiltonian is $H=\frac{1}{2}\omega \sigma _{z}$ in Eq. (20), which is the same as that of the Hamiltonian in the pure qubit. And the Lindblad operator $L_k$ satisfies $L_k=\sqrt{\gamma }\sigma _{-}$, where $\sigma _{-}=\vert 1\rangle \langle 0\vert$ is the atomic lowering operator, and $\gamma > 0$ is the rate of spontaneous emission. The Lindblad form master equation for this process can be written as

21 $$\begin{aligned} \frac{d\rho }{d t}=-i[H, \rho ]+\gamma \left[ 2 \sigma _{-}\rho \sigma _{-}^{\dagger }-\sigma _{-}^{\dagger } \sigma _{-} \rho -\rho \sigma _{-}^{\dagger } \sigma _{-}\right] . \end{aligned}$$

From Eqs. (5), (8), (10), (11) and (21), the energy change of the LGI in Eq. (8) and $K_{LG}$ in Eq. (5) under the projective measurement, can be respectively obtained as

22 $$\begin{aligned} \Delta E= & {} \frac{1}{96}e^{-4 \gamma \tau -2 i\tau \omega }\omega \left[ e^{-4 i\tau \omega }\left( 8 e^{6 i\tau \omega }\cos 2 \theta \left( -2 (\alpha +1)-(\alpha +1) e^{2 \gamma \tau }+3 e^{4 \gamma \tau }\right) \right. \right. \nonumber \\{} & {} \qquad \qquad \qquad +(\alpha -1)e^{3 \gamma \tau +7 i\tau \omega } \cos 4 \theta \nonumber \\{} & {} \left. -2 (2 \alpha +1)e^{6 i\tau \omega } \cos 4 \theta + e^{t (\gamma +5 i \omega )} \left( \alpha +(\alpha -1) e^{2 \gamma \tau }+(\alpha +1) e^{2 i\tau \omega }+1\right) \cos 4 \theta +2 e^{2\tau (\gamma +3 i \omega )}\right. \nonumber \\{} & {} \left. \qquad \times ( \cos 4 \theta (1-\alpha ) -3 \alpha +\alpha (\cos 4 \theta -1) \cos 2\omega \tau -5)\right) +4 e^{2\tau (\gamma +i \omega )} \nonumber \\{} & {}  \qquad \qquad \qquad \times \cosh \gamma \tau (19 \sinh \gamma \tau -\alpha \cos \omega \tau )\nonumber \\{} & {} \left. +2 e^{2\tau (\gamma +i \omega )} \left( 2 \sinh \gamma \tau (30 \alpha \cosh \gamma \tau +\cos \omega \tau )+(18 \alpha +5) \cosh 2 \gamma \tau \right) \right] , \end{aligned}$$

23 $$\begin{aligned} K_{LG}= & {} \frac{1}{2} e^{-2\tau (\gamma +i \omega )} \left[ \sin ^2\theta \left( 2 (1+e^{2 i\tau \omega }) e^{\tau (\gamma +i \omega )}-e^{4 i\tau \omega }-1\right) +2 e^{2\tau (\gamma +i \omega ) } \cos ^2\theta \right] . \end{aligned}$$

It can be seen from Eq. (22) that when $\theta =\frac{\pi }{2}$, $\Delta E=\Delta E_{constraint}=-{{\textrm{Tr}}}[\rho ({0})H({0})]$. In this chapter, to facilitate comparison with the violation of the LGI for other systems, we suppose $\theta =\frac{\pi }{2}$, $\tau =\frac{\pi }{4\omega }$ and $\phi =\frac{\pi }{4}$ as the condition of the energy constraint, to discuss the violations of the LGI with the projective measurement and the coarsening measurement. Then, in this case of the energy constraint condition, it can be clearly found from Eq. (23) that the LGI for the dissipative qubit under the projective measurement, can be violated in the case of $\gamma \le 0.4412\omega$. And in the previous chapter, we mentioned that for a two-level system, the maximum violation value of the LGI is 1.5^32^, while for the dissipative qubit, in the energy constraint, the maximum violation value 1.5 of the LGI cannot be realized.

Next, for the coarsening measurement reference ($\delta =0$ and $\Delta \ne 0$) and the coarsening measurement final resolution ($\delta \ne 0$ and $\Delta = 0$), we obtain the LGI (in Supplementary Information), from Eqs. (1)–(3), (5), (10) and (21). And then in the energy constraint (i.e., $\theta =\frac{\pi }{2}$, $\tau =\frac{\pi }{4\omega }$ and $\phi =\frac{\pi }{4}$), we obtain the critical values of the LGI under the coarsening measurement in reference and in final resolution, for $\gamma =0, 0.05\omega , 0.1\omega , 0.2\omega , 0.3\omega , 0.4\omega , 0.5\omega , 0.6\omega , 0.7\omega , 0.8\omega , 0.9\omega , \omega$. Similarly, the critical value of the LGI refers to the point at which $K_{LG, \Delta }$ and $K_{LG, \delta }$ equal 1. And then these results of the critical values of the LGI are summarized in Table 1. It can be clearly found from Table 1 that as the the rate of spontaneous emission $\gamma$ increases, the critical value of the LGI in the energy constraint decreases, whether the measurement is the coarsening measurement reference or the coarsening measurement final resolution. In other words, the robustness of quantum violation of the LGI decreases as the value of rate of spontaneous emission $\gamma$ increases for the coarsening measurement in reference and in final resolution. In addition, form Table 1, it was discovered that the robustness of the coarsening measurement reference is more than that of the coarsening in final measurement resolution.

Next, comparing with the LGI for the pure qubit in the energy constraint under the coarsening of measurement reference, we find that the violation condition of the LGI for the pure qubit is $\Delta \le 0.4162$, which equals to that of the LGI for the dissipative qubit in the rate of spontaneous emission $\gamma = 0$. In Table 1, it shows that the critical value of the LGI for the dissipative qubit decreases with the the rate of spontaneous emission $\gamma$ increasing. Therefore, if the LGI for the qubit in the presence of dissipation with any $\gamma$, is violated, the LGI for the pure qubit must be violated. Furthermore, for the coarsening measurement in final resolution, the LGI for the pure qubit can be violated when $\delta \le 0.0795$, which is identical to that of the LGI for the dissipative qubit with $\gamma =0$. Similarly, with $\gamma$ increasing, the critical value of the LGI for the dissipative qubit also decreases. Thus, the violation of the LGI for the qubit in the presence of dissipation with any $\gamma$, implies the violation of the LGI for the pure qubit, under the coarsening measurement in final resolution. In a word, under the energy constraint, the LGI for the dissipation qubit with any $\gamma$, can be violated for a narrower parameter than the LGI for the pure qubit, when the coarsening measurement is in reference and in final resolution. In addition, the coarsening measurement reference is more robust than that of the coarsening in final measurement resolution, which is similar to the pure qubit. Then, for the projective measurement, we find that in the energy constraint, the LGI can not attain its maximum violation value 1.5 for the dynamics under dissipation, which differs from that of the pure qubit.

### A qubit with dephasing

We consider the second case, i.e., the Hamiltonian $H=\frac{1}{2}\omega \sigma _{z}$, and the Lindblad operator $L_k=\sqrt{\gamma }\sigma _{z}$. The Lindblad form master equation for this process can be given by

24 $$\begin{aligned} \frac{d\rho }{d t}=-i[H, \rho ]+\gamma \left[ 2 \sigma _{z}\rho \sigma _{z}^{\dagger }-\sigma _{z}^{\dagger } \sigma _{z} \rho -\rho \sigma _{z}^{\dagger } \sigma _{z}\right] . \end{aligned}$$

Next, from Eqs. (5), (8), (10), (11) and (24), we obtain the energy change of the LGI in Eq. (8) and $K_{LG}$ in Eq. (5) under the projective measurement, which can be seen in Supplementary Information. It can be clearly found from Eq. (A7) that when $\theta =\frac{\pi }{2}$, $\Delta E=\Delta E_{constraint}=-{{\textrm{Tr}}}[\rho ({0})H({0})]$. Similarly, in order to facilitate comparison with the violation of the LGI for other systems (for pure, driven and dissipative qubits), in this chapter, we also consider $\theta =\frac{\pi }{2}$, $\tau =\frac{\pi }{4\omega }$ and $\phi =\frac{\pi }{4}$ as the the energy constraint condition, to discuss the quantum violations of the LGI for the dephasing qubit under the projective and coarsening measurements. In this condition of the energy constraint and the projective measurement, we find that if $\gamma \le 0.1103\omega$, the LGI for the dephasing qubit can be violated. And in the energy constraint, we obtain from Eq. (A8) that the LGI cannot be realized the maximum violation value 1.5.

From Eqs. (1)–(5), (10) and (24), the LGI for the coarsening measurement reference ($\delta =0$ and $\Delta \ne 0$) and the coarsening measurement final resolution ($\delta \ne 0$ and $\Delta = 0$), can be obtained, which is shown in Supplementary Information. Similarly, in the energy constraint, for $\gamma =0, 0.05\omega , 0.1\omega , 0.2\omega , 0.3\omega$, $0.4\omega , 0.5\omega , 0.6\omega , 0.7\omega , 0.8\omega , 0.9\omega , \omega$, the critical values of the LGI with the coarsening measurement in reference and in final resolution, can be obtained and then listed in Table 1. In Table 1, it shows that in the energy constraint, the critical value of the LGI decreases with the the rate of spontaneous emission $\gamma$ increasing, whether the way of measurement is the coarsening reference of measurement or the coarsening final resolution of measurement. Furthermore, the robustness of the coarsening measurement in final resolution is more vulnerable than that of the coarsening measurement in reference (see Table 1).

Next, in the energy constraint and the coarsening of measurement reference, comparing the LGI for the pure qubit with the dephasing qubit, we find that in the case of $\gamma = 0$, the violation condition of the LGI for these two system is the same, i.e., $\Delta \le 0.4162$. And the critical value of the LGI for the dephasing qubit decreases as the rate of spontaneous emission $\gamma$ increases (see Table 1), and therefore when the LGI for the pure qubit is satisfied, the LGI for the dephasing qubit must be satisfied too. For the coarsening measurement in final resolution, the critical value of the LGI for the pure and dephasing qubits ($\gamma = 0$) is the same. Similarly, for the dephasing qubit, with the rate of spontaneous emission $\gamma$ increasing, the critical value of the LGI also decreases. Therefore, when the pure qubit is satisfied, the LGI for the dephasing qubit must be satisfied, under the coarsening measurement in final resolution. In a word, when the coarsening measurement is at reference and at final resolution, in the case of the energy constraint, the LGI for the dephasing qubit with any $\gamma$, can be violated for a narrower parameter than that of the pure qubit. In addition, for any fixed $\gamma$, the LGI with the coarsening final measurement resolution can be violated for a narrower parameter than that of the coarsening measurement reference.

**Figure 2:**
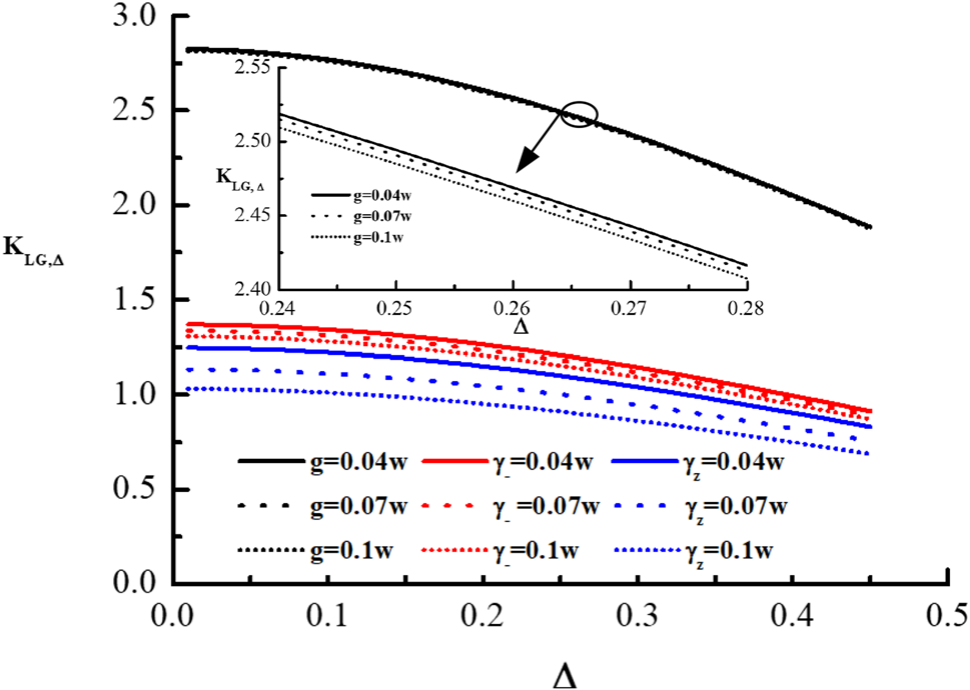
$K_{LG, \Delta }$ as a function of $\Delta$ in coarsenging measurement reference for three different values of *g* for the drive qubit (black solid line for $g=0.04\omega$, black dashed line for $g=0.07\omega$, and black short-dashed line for $g=0.1\omega$), three different values of $\gamma$ for the dissipative qubit (red solid line for $\gamma =0.04\omega$, red dashed line for $\gamma =0.07\omega$, and red short-dashed line for $\gamma =0.1\omega$), and three different values of $\gamma$ for the dephasing qubit (blue solid line for $\gamma =0.04\omega$, blue dashed line for $\gamma =0.07\omega$, and blue short-dashed line for $\gamma =0.1\omega$). For the sake of clarity, we have included a small graph in the figure that illustrates the different values of g in the driven system. The small graph displays three different lines: a black solid line representing $g=0.04\omega$, a black dashed line representing $g=0.07\omega$, and a black short-dashed line representing $g=0.1\omega$.

**Figure 3:**
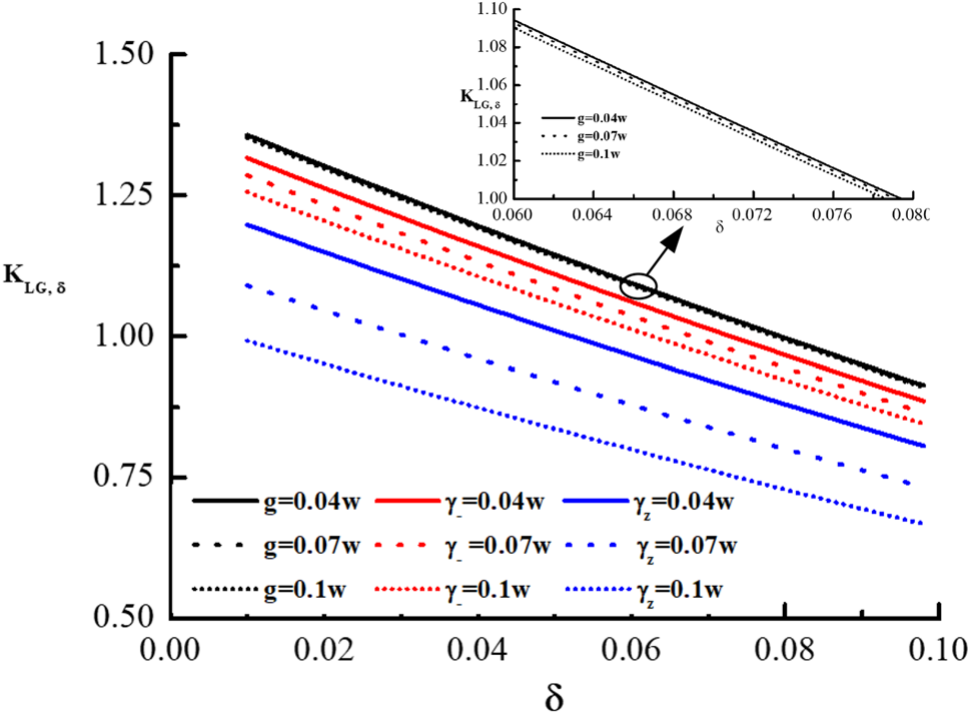
$K_{LG, \delta }$ as a function of $\delta$ in the coarsening measurement final resolution for three different values of *g* for the drive qubit (black solid line for $g=0.04\omega$, black dashed line for $g=0.07\omega$, and black short-dashed line for $g=0.1\omega$), three different values of $\gamma$ for the dissipative qubit (red solid line for $\gamma =0.04\omega$, red dashed line for $\gamma =0.07\omega$, and red short-dashed line for $\gamma =0.1\omega$), and three different values of $\gamma$ for the dephasing qubit (blue solid line for $\gamma =0.04\omega$, blue dashed line for $\gamma =0.07\omega$, and blue short-dashed line for $\gamma =0.1\omega$). Similarly, to improve clarity, we have included a small graph within the figure, which illustrates three different values of $g$ for the driven system. This small graph features three separate lines: a solid black line indicating $g=0.04\omega$, a dashed black line indicating $g=0.07\omega$, and a short-dashed black line indicating $g=0.1\omega$.

Then, in order to compare the violation condition of the LGI in the energy constraint for the driven, dissipation and dephasing qubits, under the coarsening measurement reference, we plot Fig. 2. It shows that the LG function $K_{LG, \Delta }$ as a function of $\Delta$ in the coarsening measurement reference with $g =0.04\omega , 0.07\omega , 0.1\omega$ for driven qubit, and $\gamma =0.04\omega , 0.07\omega , 0.1\omega$ for dissipative and dephasing qubits. It can be found from Fig. 2 that as g and $\gamma$ increase, the critical values of the LGI for the driven, dissipation and dephasing qubits decrease, respectively, while the increment increases. And for any fixed g and $\gamma$, the robustness of quantum violation of the LGI for the system with driven is the most robust, which is more robust than that of the qubit with dephasing, and the robustness of the qubit with dissipation lies between the driven and dephasing qubits, for the coarsening measurement reference. In Fig. 2, it also shows that $K_{LG, \Delta }$ decreases as the coarsening degree in measurement reference $\Delta$ increases. Similarly, for the coarsening final measurement resolution, we plot the LG function $K_{LG, \delta }$ as a function of $\delta$ in Fig. 3 to compare the violation of the LGI for the driven, dissipation and dephasing qubits. The Fig. 3 is conducted for energy constraint and different values of *g* ($0.04\omega$, $0.07\omega$, and $0.1\omega$) for the driven qubit, and $\gamma$ ($0.04\omega$, $0.07\omega$, and $0.1\omega$) for dissipative and dephasing qubits. In Fig. 3, it can be clearly found that the critical values of the LGI for the driven, dissipation and dephasing qubits decrease with g and $\gamma$ increasing, respectively, while the increment increases. Furthermore, in the case of any fixed g and $\gamma$, quantum violation of the LGI for the dephasing qubit is the most vulnerable, and the dissipative qubit lies between the driven and dephasing qubits, which is more vulnerable than that of the qubit with driven for the coarsening measurement resolution. From Fig. 3, we also find that with the coarsening degree in measurement final resolution $\delta$ increasing, $K_{LG, \delta }$ decreases. In addition, for dephasing qubit, we find that the coarsening final resolution of measurement is more vulnerable than that of the coarsening reference of measurement, which is similar to that of the pure coherent dynamics, dynamics with drive and dynamics under dissipation (see Table 1). Furthermore, in the energy constraint and the projective measurement, we find that the LGI can not attain its maximum violation value 1.5 for the dynamics under dephasing, which is the same as that of the drive and dissipative qubits, and is different from that of the pure qubit.

## Discussion

In this paper, we examine the LGI for a qubit in following different four scenarios: with drive, without drive, in the presence of dissipation, and in the presence of dephasing. Two measurement methods are considered: the projective measurement and the coarsening measurement, and the measurement of coarsening is categorized as coarsening measurement reference, coarsening measurement final resolution and coarsening measurement time reference. We explore the quantum violation of the LGI in an energy constraint (i.e., the energy change of the LGI with the projective measurement is equal to the negative of the energy of the initial state) for these different scenarios systems. In the energy constraint, for the projective measurement, the coarsening measurement reference and the coarsening measurement final resolution, we studied the effects of them on the LGI with pure, driven, dissipative and dephasing qubits; and for the coarsening measurement tenmporal reference, we only discuss the effects of it on the LGI with pure and driven qubits. When the coarsening measurement reference and the coarsening final measurement resolution is coarsened, we find that in the energy constraint, for the qubit with drive, dissipation and dephasing, as the driven intensity g and the rate of spontaneous emission $\gamma$ increase, the critical values of the LGI for these three scenarios systems decrease, respectively (see Table 1). And for any fixed g and $\gamma$, the robustness of quantum violation of the LGI for the system with driven is more robust than that of the qubit with dissipation, and the qubit with dissipation is more robust than that of the qubit with dephasing, which are both more vulnerable than that of the pure qubit, for the coarsening measurement in reference and in final resolution. That is to say, in the case of the energy constraint, the robustness of quantum violation of the LGI for the dephasing qubit is more vulnerable than that of the dissipation qubit, which is more vulnerable than that of the system with driven, and the quantum violation of the LGI for the coherent dynamics is the most robust, for the coarsening measurement reference and the coarsening final measurement resolution. Physically, this phenomenon might be understood as following: the LGI is related to coherence, and quantum coherence is the reason why the assumption of realism has to be rejected^14^. Furthermore, quantum coherence is fragile due to coupling with the external field and the environment. For the driven, dissipation and dephasing qubits, they couple to the external field and the environment, and therefore, the robustness of quantum violation of the LGI for these scenario systems will become vulnerable. Moreover, when the temporal reference of the measurement is coarsened, in the energy constraint, the critical value of the LGI for the qubit with driven decreases with the driven intensity g increasing. And the robustness of quantum violation of the LGI for the system with driven in the energy constraint, is more vulnerable than that of the pure qubit under coarsening measurement temporal reference. Similarly, the reason of above phenomenon might be that the system with driven couples to the external field, which makes the robustness of quantum violation of the LGI become vulnerable. In addition, when subjected to an energy constraint, for projective measurement, the LGI exhibits a maximum violation value of 1.5 for coherent dynamics, but fails to reach this value (1.5) for dynamics with drive, dissipation, and dephasing. The energy change of the LGI does depend on the initial state, which differs from the LGI. Comparing critical values of the LGI (see Table 1), we find that for the system in the presence of dissipation and dephasing, the coarsening measurement reference can be violated for a wider parameter than that of the coarsening measurement in final resolution, in the energy constraint; while for the coherent dynamics and the system with drive, the coarsening of measurement in time reference can be violated for a wider parameter than that of the coarsening measurement reference, and the relationship among the coarsening measurement reference and the coarsening measurement in final resolution is similar to that of the system in the presence of dissipation and dephasing. Furthermore, in this paper, we consider three particular types of coarsening measurement, i.e., the coarsening in measurement reference, in final measurement resolution and in measurement time reference. However, there are other types of coarsening measurements, and we guess that the conclusions of this paper might be also applicable to other types of coarsening measurements, but it requires further investigation. In future research, we will continue to pay attention to this issue and hope to discover a more comprehensive proof for the logical correlation between the energy constraint and the LGI for different systems and measurement methods.

## Supplementary Information

Supplementary Information.

## Data Availability

All data generated or analysed during this study are included in this published article and its supplementary information files.
