# Supplementary material for: Quantum violation of LGI under an energy constraint for different scenarios systems
Source: Sci Rep. 2023 Aug 2;13:12530. doi: 10.1038/s41598-023-39612-6 (PMC10397236; doi:10.1038/s41598-023-39612-6)
Supplement: Supplementary file 1 — Supplementary Information. [file 41598_2023_39612_MOESM1_ESM.pdf]

Quantum violation of LGI under an energy constraint for different scenarios systems

Yuxia Zhang [*]   Xiangguan Tan   Tianhui Qiu

A A Qubit with Driven

For the projective measurement, the coarsening measurement reference, the coarsening measurement in final resolution and coarsening measurement temporal reference, using Eqs. (1-5), (10), (11), (13), (14), (17) and (18), the LGI in the energy constraint can be respectively given as

$$K_{LG} = \frac{1}{2}\left[\cos\left(\frac{g\pi}{2\omega}\right) + 2\sqrt{2} - 1\right], \tag{A1}$$

$$K_{LG,\Delta} = e^{-2\Delta^2}\left[\sqrt{2} - \sin^2\left(\frac{\pi g}{4\omega}\right)\right], \tag{A2}$$

$$K_{LG,\delta} = (1-2\delta)^2\left[\sqrt{2} - \sin^2\left(\frac{\pi g}{4\omega}\right)\right], \tag{A3}$$

$$\begin{aligned} K_{LG,\Delta'} = \frac{1}{8}(-1)^{3/4} e^{\left(-\frac{9\Delta'^2\omega^2+4\Delta'^2g^2+8\Delta'^2g\omega+i\pi g\omega}{2\omega^2}\right)} \Bigg(& -i\sqrt{2}e^{\frac{5\Delta'^2}{2}} - \sqrt{2}e^{\frac{1}{2}\Delta'^2\left(\frac{16g}{\omega}+5\right)} - \sqrt{2}e^{\left(\frac{5\Delta'^2}{2}+\frac{i\pi g}{\omega}\right)} - i\sqrt{2}e^{\left(\frac{5\Delta'^2}{2}+\frac{8\Delta'^2 g}{\omega}+\frac{i\pi g}{\omega}\right)} \\ & + e^{\frac{g\left(4\Delta'^2\omega+6\Delta'^2g+i\pi\omega\right)}{4\omega^2}} - (2+2i)e^{\frac{g\left(8\Delta'^2\omega+4\Delta'^2g+i\pi\omega\right)}{2\omega^2}} + e^{\frac{g\left(28\Delta'^2\omega+6\Delta'^2g+3i\pi\omega\right)}{4\omega^2}} + ie^{\frac{g\left(4\Delta'^2\omega+6\Delta'^2g+3i\pi\omega\right)}{4\omega^2}} + ie^{\frac{g\left(28\Delta'^2\omega+6\Delta'^2g+i\pi\omega\right)}{4\omega^2}} \\ & - (2-i)e^{\left(\frac{16\Delta'^2\omega^2+6\Delta'^2g^2+12\Delta'^2g\omega+i\pi g\omega}{4\omega^2}\right)} - (6+6i)e^{\left(\frac{8\Delta'^2\omega^2+4\Delta'^2g^2+8\Delta'^2g\omega+i\pi g\omega}{2\omega^2}\right)} + (1-2i)e^{\left(\frac{16\Delta'^2\omega^2+6\Delta'^2g^2+12\Delta'^2g\omega+3i\pi g\omega}{4\omega^2}\right)} \\ & + (2+2i)\sqrt{2}e^{\left(\frac{5\Delta'^2\omega^2+4\Delta'^2g^2+8\Delta'^2g\omega+i\pi g\omega}{2\omega^2}\right)} + (1-2i)e^{\left(\frac{16\Delta'^2\omega^2+6\Delta'^2g^2+20\Delta'^2g\omega+i\pi g\omega}{4\omega^2}\right)} - (2-i)e^{\left(\frac{16\Delta'^2\omega^2+6\Delta'^2g^2+20\Delta'^2g\omega+3i\pi g\omega}{4\omega^2}\right)}\Bigg). \end{aligned} \tag{A4}$$

*zhangyuxia0619@163.com

From Eq. (A1), we find that the LGI for the projective measurement can be violated (i.e., Eq. (5) can be violated), when $0 \le g \le 0.8902\omega$. When the measurement reference, the measurement final resolution and measurement reference in time are coarsened, from Eqs. (A2) and (A4), it can be found the critical values of the LGI for the driven qubit. These critical values obtained for the LGI in these situation are summarized in Table 1.

B A Qubit with Dissipation

Using Eqs. (1-3), (5), (10) and (21), the LGI for the coarsening measurement in reference and the coarsening measurement in final resolution, can be respectively expressed as

$$K_{LG,\Delta} = \frac{1}{2}e^{-2\left(\Delta^2+\tau(\gamma+i\omega)\right)}\left[2\cos^2\theta e^{\Delta^2+2\gamma\tau+2i\tau\omega} + \sin^2\theta\left(2(1+e^{2i\omega\tau})e^{\tau(\gamma+i\omega)} - e^{4i\omega\tau} - 1\right)\right], \tag{A5}$$

$$K_{LG,\delta} = \frac{1}{2}(1-2\delta)^2 e^{-2\tau(\gamma+i\omega)}\left[\sin^2\theta\left(2e^{\tau(\gamma+i\omega)}(1+e^{2i\omega\tau}) - e^{4i\tau\omega} - 1\right) + 2e^{2\tau(\gamma+i\omega)}\cos^2\theta\right]. \tag{A6}$$

From Eqs. (A5) and (A6), we find the critical values of the LGI in the case of the energy constraint (i.e., $\theta = \frac{\pi}{2}$, $\tau = \frac{\pi}{4\omega}$ and $\phi = \frac{\pi}{4}$), for the coarsening in measurement reference and in final resolution, and then summarize them in Table. 1.

C A Qubit with Dephasing

From Eqs. (1-3), (5), (8), (10), (11) and (24), the energy change of the LGI in Eq. (8) and K_{LG} in Eq. (5) for the projective measurement, the coarsening measurement reference and the coarsening measurement in final resolution, can be respectively obtained as

$$\Delta E = \frac{1}{4}\alpha\omega\sin^2\theta e^{-8\gamma\tau-2i\omega\tau}\left[3(\cos 2\theta+3)e^{8\gamma\tau+2i\omega\tau} - \cos^2\theta\left(2\left(1+e^{2i\omega\tau}\right)e^{4\gamma\tau+i\omega\tau} + e^{4i\omega\tau} + 1\right)\right], \tag{A7}$$

$$K_{LG} = \cos^2\theta + \sin^2\theta e^{-8\gamma\tau}\left(2e^{4\gamma\tau}\cos\omega\tau - \cos 2\omega\tau\right), \tag{A8}$$

$$K_{LG,\Delta}=\frac{1}{2}e^{-2\left(\Delta^2+4\gamma\tau+i\omega\tau\right)}\left[2\cos^2\theta e^{\Delta^2+8\gamma\tau+2i\omega\tau}+\sin^2\theta\left(2(1+e^{2i\omega\tau})e^{4\gamma\tau+i\omega\tau}-e^{4i\omega\tau}-1\right)\right], \tag{A9}$$

$$K_{LG,\delta}=-\frac{1}{2}(1-2\delta)^2e^{-8\gamma\tau-2i\omega\tau}\left[\sin^2\theta\left(-2(1+e^{2i\omega\tau})e^{4\gamma\tau+i\omega\tau}+e^{4i\omega\tau}+1\right)-2\cos^2\theta e^{8\gamma\tau+2i\omega\tau}\right]. \tag{A10}$$

It can be clearly from Eq. (A7) found that if $\theta=\frac{\pi}{2}$, $\Delta E=\Delta E_{constraint}=-\mathrm{Tr}[\rho(0)H(0)]$. It is noted that in this chapter, we will use $\theta=\frac{\pi}{2}$, $\tau=\frac{\pi}{4\omega}$ and $\phi=\frac{\pi}{4}$ as the energy constraint condition to investigate the quantum violation of the LGI for the dephasing qubit, under projective and coarsening measurements. Then, from Eq. (A8), we find that the LGI for the projective measurement in the case of the energy constraint, can be violated with $\gamma\leq 0.1103\omega$. In addition, from Eq. (A8), we find that in the energy constraint, the LGI cannot be realized the maximum violation value 1.5. Then, from Eqs. (A9) and (A10), it can be found the critical values of the LGI in the energy constraint (i.e., $\theta=\frac{\pi}{2}$, $\tau=\frac{\pi}{4\omega}$ and $\phi=\frac{\pi}{4}$), for the coarsening in measurement reference and in final resolution, and then we summarize them in Table. 1.
